# Linking Eye Design with Host Symbiont Relationships in Pontoniine Shrimps (Crustacea, Decapoda, Palaemonidae)

**DOI:** 10.1371/journal.pone.0099505

**Published:** 2014-06-20

**Authors:** Nicola C. Dobson, Sammy De Grave, Magnus L. Johnson

**Affiliations:** 1 Centre for Environmental and Marine Sciences, Faculty of Science and Engineering, University of Hull, Scarborough Campus, Scarborough, North Yorkshire, United Kingdom; 2 Oxford University Museum of Natural History, Oxford, United Kingdom; Academia Sinica, Taiwan

## Abstract

Symbiosis is prevalent in the marine environment with many studies examining the effects of such interactions between host and symbiont. Pontoniine shrimps are a group whose ecology is characterised by symbiotic interactions. This investigation examines the gross morphology of Pontoniinae compound eyes and superficial optical parameters with reference to their symbiotic relationship or lifestyle category; free-living, ectosymbiont, endosymbiont (bivalves) or endosymbiont (non-bivalves). The eye morphologies of free-living and ectosymbiotic species are very similar, yet differ from both forms of endosymbiotic species. Endosymbionts have significantly smaller and simpler eyes with larger facets and bigger interommatidial angles and eye parameters for increased sensitivity levels. However bivalve endosymbionts form an intermediary group between non-bivalve endosymbionts and ectosymbionts as a result of their more active lifestyle. The accessory eye or “nebenauge”, although of uncertain function, commonly occurs in free-living Pontoniinae species but rarely in endosymbionts apart from in more primitive species. The variation in morphology reflects tensions between functional requirements and ecological pressures that have strongly influenced eye design in Pontoniinae.

## Introduction

Symbiotic interactions are found throughout the marine environment with relationships providing sources of shelter, reproduction opportunities, food and nutrition [1]. Symbioses are generally categorised as mutualistic, commensal or parasitic depending on the costs and benefits of the association, the level of dependency on the host (facultative or obligate) and the number of taxa/species they associate with (generalist or specialist) [2], [3].

Symbionts can also be characterised by their mode of association or ‘lifestyle’, defined as either ectosymbionts, populating the external surface of their host or endosymbionts residing within their host [4], [5]. This type of classification follows a more general idea of associations, as often the type of association (mutualistic, parasitic etc.) is not known for many species [6]. Crustaceans including many crab, amphipod, isopod and shrimp species are symbiotic with a whole spectrum of host taxa [1], [7], [8]. Within Caridean shrimps, most of these associations occur in Pontoniinae (a subfamily of Palaemonidae) [6], [9]–[12] and to a lesser extent Alpheidae, but are also known for a few taxa in other families (e.g. Hippolytidae). Although these associations in Pontoniinae were traditionally classed as commensal, Ďuriš *et al*., [13] have suggested this may not be valid for all species, and some are perhaps better considered as parasites.

Currently there are approximately 602 recognised species of Pontoniinae [14] which have their centre of diversity in tropical and subtropical regions [15]–[17]. They reach peak biodiversity on Indo-Pacific coral reefs [6], [9], [18], with fewer species found in the Caribbean [19], [20]. Most species inhabit shallow coastal waters [21], with the majority of species within the first 100 m (Appendix S1), although *Periclimenes pholeter* has been reported from waters in excess of 2000 m [22]. Pontoniid shrimps are known for their cryptic behaviour and symbiotic associations with a range of host taxa including *inter alia* bivalves, corals, anemones, sponges and jellyfish [18], [23], [24] and several species act as fish cleaners [16], [25]. Approximately 60–70% of Pontoniinae are symbiotic [18]; however this estimation is likely an underestimate, as this is unknown for numerous taxa. Species that do not form associations with other host taxa are regarded as free-living [26]–[28], for example micro-predators found in seagrass meadows [6], [27], [29], [30]. The association of Pontoniinae with numerous taxa has resulted in the evolution of morphological adaptations [21], [31] and adaptive radiation [32]. Free-living species often have a general palaemonid bauplan, with a well-developed dentate rostrum and long slender chelae and pereiopods [6], [33]. Morphological modification of the pontoniid bauplan is however extensive, with significant departures in general body shape, rostrum, mouthparts and ambulatory legs. For instance, *Ischnopontonia lophos* is laterally flattened with highly jointed chelipeds, an adaptation to living in-between elongated *Galaxea* corallites [34]. Other ectosymbiont coral associates (e.g. *Coralliocaris* spp. and *Jocaste* spp.) possess modified walking legs with a grasping dactyl to enhance their grip on their coral host [29], [35]. Sponge-dwelling endosymbionts (e.g. *Apopontonia*, *Typton)* are often small to medium sized, sometimes with a swollen carapace and frequently with a diminutive rostrum [6], [36]. *Typton* species also have shearing-type chelae for feeding on host tissue, and are now regarded as parasitic [13]. Species from the genera *Dactylonia*, *Odontonia* and *Ascidonia* are ascidian endosymbionts [1], [37]. Differences in the bauplan of these endosymbionts are partially driven by the colonial or solitary nature of the ascidian host [15]. Unlike sponge and ascidian-dwelling endosymbionts, bivalve endosymbionts are usually larger bodied, frequently with symmetrical chelae. These species are normally found in heterosexual pairs located within the mantle cavity of the mollusc [3], [38]. Ectosymbiotic echinoid associates (e.g. *Tuleariocaris,* S*tegopontonia*) provide a further variant and possess extremely short, stout pereiopods used to hold the echinoid spines [39]. Further morphological adaptations, such as the grasping structures on the dactyli and propodi (the last two segments on the pereiopods), observed for these symbionts show morphological convergence in response to host association and can be seen in both related and unrelated species [15], [40]. Despite numerous morphological adaptations in general bauplan, the adaption of pontoniid eyes to hosts has not been considered in spite of noticeable variations in the morphology and as documented in taxonomic descriptions.

For crustaceans the ancestral eye is believed to have consisted of eyes mounted on stalks [41], [42] and is considered an adaptation of motile animals [6]. Although apposition type eyes are believed to represent the plesiomorphic condition, superposition compound eyes are the most abundant form for extant adult decapods [43]; however maintaining sensory and neural systems is metabolically costly [44]. Many studies have demonstrated how compound eyes exhibit morphological variation as a result of habitat requirements and behaviour [45]. Large differences can be seen in the tapetal distribution around the eyes of mesopelagic species depending on their depth distribution [46], [47]. Interommatidial angles [48], [49], spectral sensitivity [50], [51] and visual acuity [52] also vary with depth in pelagic crustaceans. Some early studies reported the degeneration of eyes in deep water species [53], similar to those described in troglobitic species [54], whilst other deep water species possess well-developed fully functioning eyes [48], [49], [53], [55]–[57]. Vision is regarded as playing an important role for decapods in host/shelter location, predator detection [58], orientation [59], [60] and aggressive interactions between conspecifics [61], [62]. Laboratory experiments using the symbiotic shrimp *Gnathophylloides mineri*, with or without the use of chemical cues, demonstrated that these shrimps actively seek their specific host using visual cues [63]. To date the use of visual cues for locating hosts has not been tested for Pontoniinae.

Variations in the external morphology of compound eyes have received less attention than their internal structures but such information may be useful in understanding the ecology of an animal. This is especially true for compound eyes where superficial external features are directly related to optical performance [64]. For example facet diameter, eye diameter, interommatidial angle (Δφ) and eyestalk dimensions have implications for visual function. Increased photon catch can be achieved by increasing facet diameter or increasing Δφ, thus enhancing sensitivity. However, an increasing resolution can be achieved by increasing eye diameter or decreasing Δφ [65]. As such there is a compromise between sensitivity and acuity in response to the visual requirements of the organism [64], [66].

Alpha level taxonomic species descriptions often note the presence or absence of an accessory eye spot. The accessory eye spot, ‘ocelli’, [67] or ‘dorsal spot’ and hereafter referred to as the nebenauge, is a collection of pigmented cells peculiar to caridean shrimps, but for which the function is unknown [46], [53], [67] and is integrated with or adjacent to the cornea [53], [67]. Histological examination of eyes from mesopelagic shrimp species suggests that the nebenauge could detect light [46].

The gross morphology of Pontoniinae eyes was examined in comparison to the lifestyle category of these shrimps to determine whether species living in complex visual habitats, such as free-living or ectosymbiotic species, would have features indicative of good resolution and sensitivity. In contrast, species living in less visually complex habitats, such as endosymbiotic species, could potentially possess more rudimentary, less energetically costly eyes. Although the function of the nebenauge remains unknown, due to the presence of light detecting features noted by Gaten *et al*. [46] in other decapod species, we speculate that the nebenauge should be more prevalent in species that are active in brighter and more complex habitats.

## Materials and Methods

A total of 96 Pontoniinae species from shallow water habitats were examined from 40 genera (Appendix S1). The work described in this paper was reviewed and approved by the Department of Biological Sciences, Faculty of Sciences ethics committee. Species were categorised as free-living, ectosymbionts or endosymbionts based on their listed symbiotic association or absence thereof in primary taxonomic literature (e.g. [26], [68]). Preliminary analysis revealed consistent differences in eye diameter, facet diameter and interommatidial angles between bivalve and non-bivalve endosymbionts, and thus these two categories are used throughout.

Specimens were examined using a binocular dissecting microscope with a calibrated ocular micrometer. Five external features of eye dimensions (eye diameter (ED), total eye and eyestalk length (TEASL), eyestalk length (ESL), facet diameter (FD) and nebenauge diameter (ND) in addition to post orbital carapace length) were recorded (Fig. S1 and S2). Measurements of FD and ND were determined from digital photographs using Scion Image (Scion Corporation). Five facets were measured per specimen from the central region of the cornea to determine average facet diameter. The final optical characteristic recorded was interommatidial angle (Δφ), the angle between the axes of adjacent ommatidia [69]. As this feature cannot be measured directly from the external morphology of the eye it was estimated mathematically using Equation 1 which assumes a perfectly spherical eye (adapted from Stavenga, [70]):
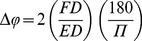
(1)


Differences in post orbital carapace lengths among the lifestyles were tested by using the Kruskal Wallis test. Optical characteristics of pontoniid eyes, such as relative eye diameter (standardised by POCL), relative nebenauge diameter (standardised by ED) and Δ*φ* (log_10_ transformed), were compared among the three lifestyles using one-way analysis of variance (ANOVA) and *Post hoc* Tukey test. Spearman’s Rank correlations were used to determine relationships between ED and FD in addition to between both ED and TEASL in relation to POCL. As the data violated the assumption of independence ANCOVA analysis could not be performed [71]. As an alternative, differences in relative TEASL (standardised by POCL) were ascertained among the lifestyles using Kruskal Wallis. Spearman’s Ranks correlations were also tested for relationships between eye stalk length (ESL) and facet diameter (FD) but since FD scaled to ED and ESL to CL, both variables were standardised correspondingly prior to analysis. Eye parameter (EP) was additionally calculated as a measure of quantifying the trade-off between resolution and sensitivity of the eyes [72] based on Snyder’s [73] equation:
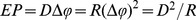
(2)


Eye parameter can be calculated in 3 different ways (Equation 2) using a combination of facet diameter (D µm), interommatidial angle (Δ*φ* in radians) or eye radius (R µm). In this investigation eye parameter was calculated using DΔ*φ*. Statistical differences in average EPs among the lifestyle categories were ascertained by means of the Kruskal Wallis test. A Chi-squared test for association was performed to determine whether there was an association in the occurrence of the nebenauge. All analyses were performed using the Statistical Software Package R 3.0.2 [74].

## Results

**Figure 1:**
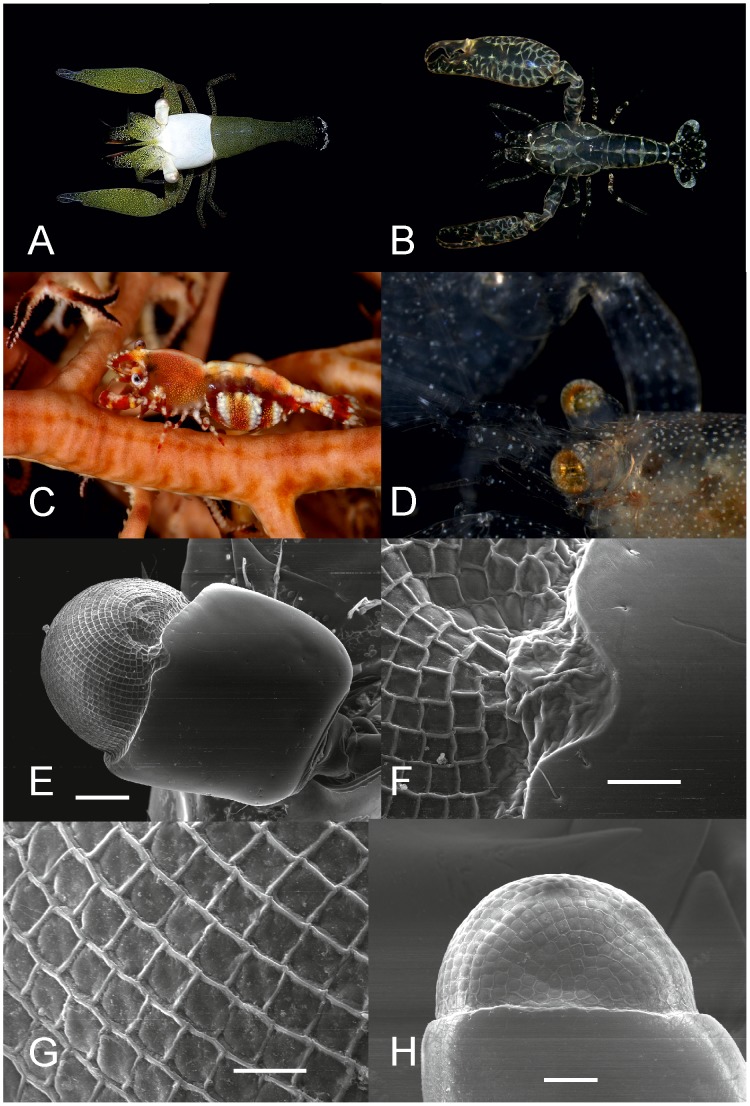
In life photographs and SEM images of pontoniine shrimps A) *Coralliocaris superba*, ectosymbiont coral associate; B) *Pontonia mexicana*, endosymbiont bivalve associate; C) *Periclimenes perryae*, ectosymbiont echinoderm associate; D) *Periclimenaeus sp*., endosymbiont sponge associate, life colouration of eyes; E) *Cuapetes americanus*, free-living species, left eye including cornea and eyestalk; F) *Cuapetes americanus* nebenauge; G) *Cuapetes americanus*, lattice facet structure; H) *Pontonia mexicana*, cornea. Scale bars indicate 100 µm (E, H) or 20 µm (F, G). Photo credits: A–D - A.Anker; E–H - S. De Grave.

Based on the species of Pontoniinae examined, variations in body size and eye morphology were observed in relation to their lifestyle (Fig. 1). Post-orbital carapace length (POCL) differed among the four lifestyles categories (Kruskal Wallis, H (adjusted for ties) = 30.82, df = 3, *P* = 0.001). *Post hoc* comparisons (*P*<0.05) revealed that POCL of endosymbionts (bivalves) (median 6.01 mm, range 4.5–10.2 mm) were significantly larger than endosymbionts (non-bivalves) (median 1.77 mm, range 1.17–5.6 mm), ectosymbionts (median 2.19 mm, range 0.9–5.5 mm) and free-living species (median 2.46 mm, range 1.38–4.13 mm), however no significant differences were found among any of the other categories.

**Figure 2:**
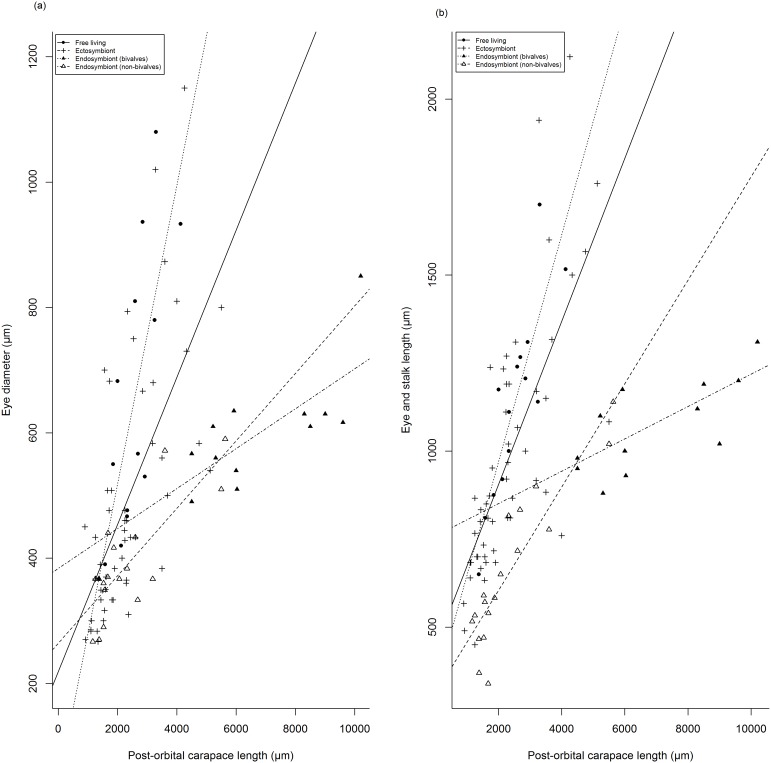
Relationship between post-orbital carapace length and a) eye diameter. b) eye and stalk length, for 96 species of Pontoniinae associated with four lifestyle categories.

**Figure 3:**
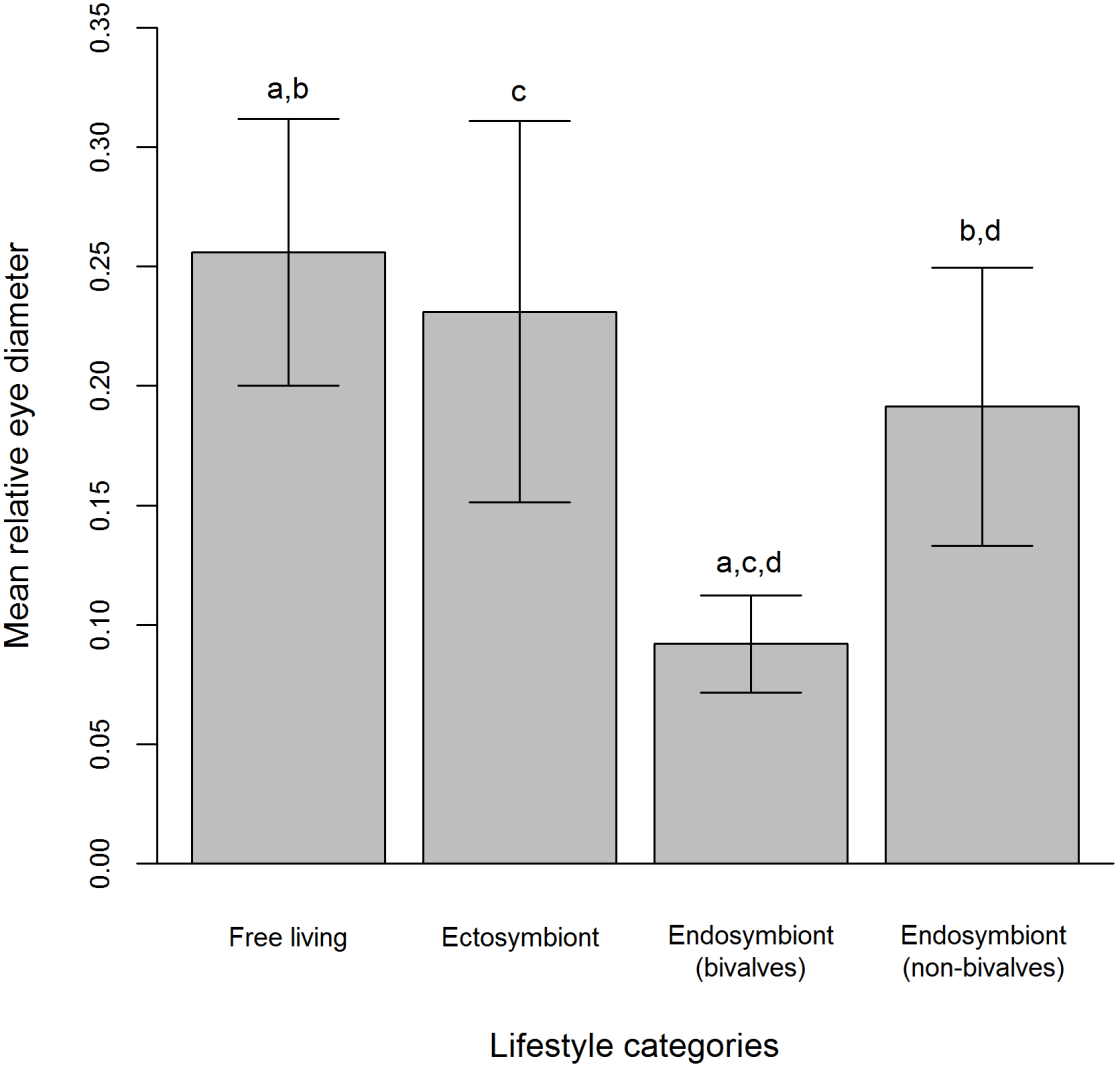
Mean relative eye diameter (standardised by post-orbital carapace length) for 96 species of Pontoniinae associated with four lifestyle categories. Significant differences (Tukey HSD *P*<0.05) between lifestyle categories were denoted as a, b, c and d, with lifestyle categories bearing the same letter being statistically different.

**Figure 4:**
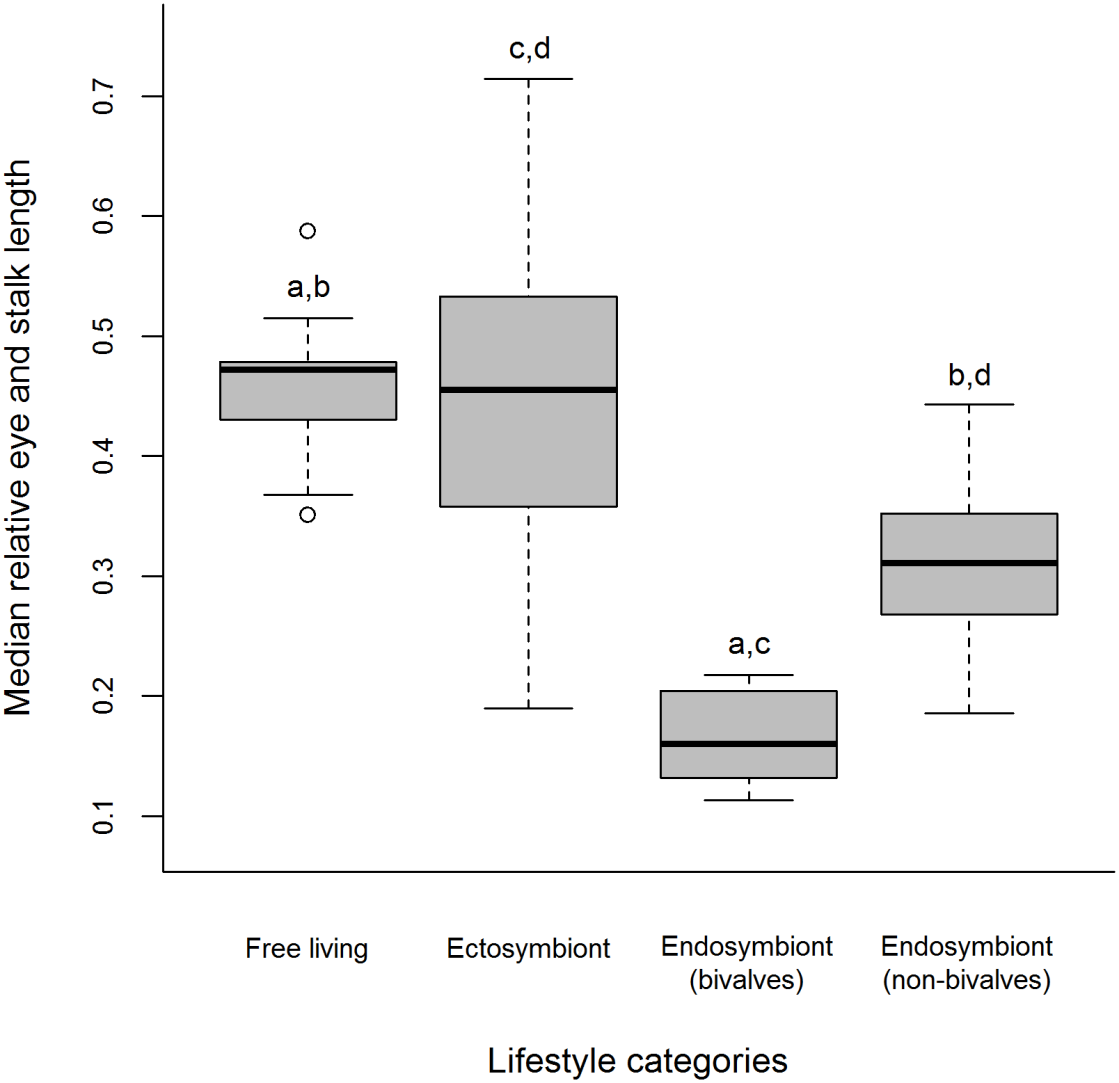
Median relative eye and stalk length (standardised by post-orbital carapace length) for 96 species of Pontoniinae associated with four lifestyle categories. Significant differences (*Post hoc* pairwise comparisons *P*<0.05) between lifestyle categories were denoted as a, b, c and d, with lifestyle categories bearing the same letter being statistically different.

**Figure 5:**
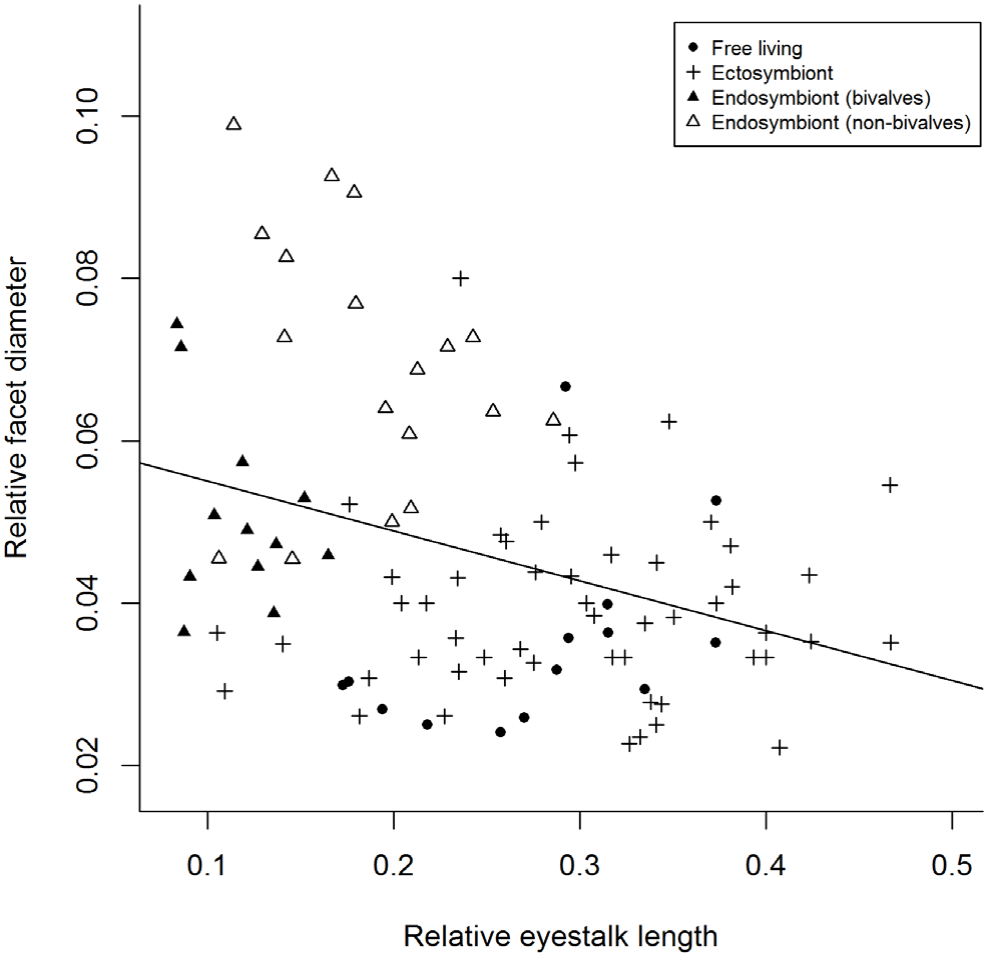
Relationship between relative eyestalk length (standardised by post-orbital carapace length) and relative facet diameter (standardised by eye diameter) for 96 species of Pontoniinae associated with four lifestyles categories.

Optical traits, ED and TEASL, are, as expected, correlated with POCL (Table 1), with larger species possessing larger eyes. However, morphological variation can be observed within these features according to their categorised lifestyles (Fig. 2a & b), with the scaling of these relationships varying among lifestyle categories. In spite of similar positive relationships between POCL against ED and TEASL, there are differences in the relative size of these features by lifestyle category. Endosymbionts (bivalves) have smaller relative eye diameters (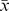 = 0.092, SD±0.02) than endosymbiont (non-bivalves) (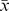 = 0.19, SD±0.06), ectosymbionts (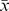 = 0.231, SD±0.08) and free-living species (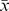 = 0.256, SD±0.06). The relative EDs of endosymbiont (non-bivalves) are also smaller than free-living species, however no additional differences in relative ED were observed among the remaining lifestyle categories (ANOVA, F_3,92_ = 16.21, *P*<0.001, Tukey *P* = 0.05) (Fig. 3). Although endosymbiont (bivalves) have smaller ED’s relative to POCL, they possess larger facet diameters relative to ED (ANOVA, F_3,92_ = 28.40, *P*<0.001, Tukey *P* = 0.05) than both ectosymbiotic (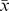 = −1.423, SD±0.12) and free-living species (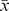 = −1.475, SD±0.12), this is illustrated by Fig. 1E & H. However endosymbiont (non-bivalves) have larger facet diameters relative to ED than any other lifestyle categories (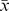 = −1.167, SD±0.10), no differences were detected between ectosymbiont and free-living species. Endosymbiont (bivalves) (median = 0.16, range 0.113–0.217) and endosymbiont (non-bivalves) (median = 0.311, range 0.186–0.443) have smaller relative eye and stalk lengths than both ectosymbionts (median = 0.455, range 0.19–0.714) and free-living species (median = 0.472, range 0.351–0.587) but are not larger or smaller than each other. Additionally no differences were observed between free-living and ectosymbionts (Kruskal Wallis, H (adjusted for ties) = 48.35, df = 3, *P*<0.001, *Post hoc* pairwise comparisons *P* = 0.05) (Fig. 4). Relative eyestalk length was found to have a negative relationship with relative facet diameter (Spearman’s rank correlation, r_s_ = −0.309, df = 96, P = 0.002) with longer eyestalks possessing eyes with smaller facet diameters (Fig. 5). The same relationship was also observed for interommatidial angle (Δφ) (Spearman’s rank correlation, r_s_ = −0.309, df = 96, P = 0.002).

**Table 1:** Spearman’s Rank Correlations, comparisons of post orbital carapace length (POCL) against eye diameter (ED) and eye and stalk length (TEASL) and relationship between ED and facet diameter (FD) for 96 species of Pontoniinae associated with four lifestyle categories.

Parameters	Free-living			Ectosymbiont			Endosymbiont (bivalves)			Endosymbiont (non-bivalves)		
	n/df	r_s_ value	P value	n/df	r_s_ value	P value	n/df	r_s_ value	P value	n/df	r_s_ value	P value
POCL vs. ED	14	0.767	0.001	52	0.706	0.001	12	0.605	0.037	18	0.696	0.001
POCL vs. TEASL	14	0.877	0.001	52	0.787	0.001	12	0.676	0.016	18	0.866	0.001
ED vs. FD	14	0.641	0.014	52	0.704	0.001	12	0.636	0.026	18	0.758	0.001

**Figure 6:**
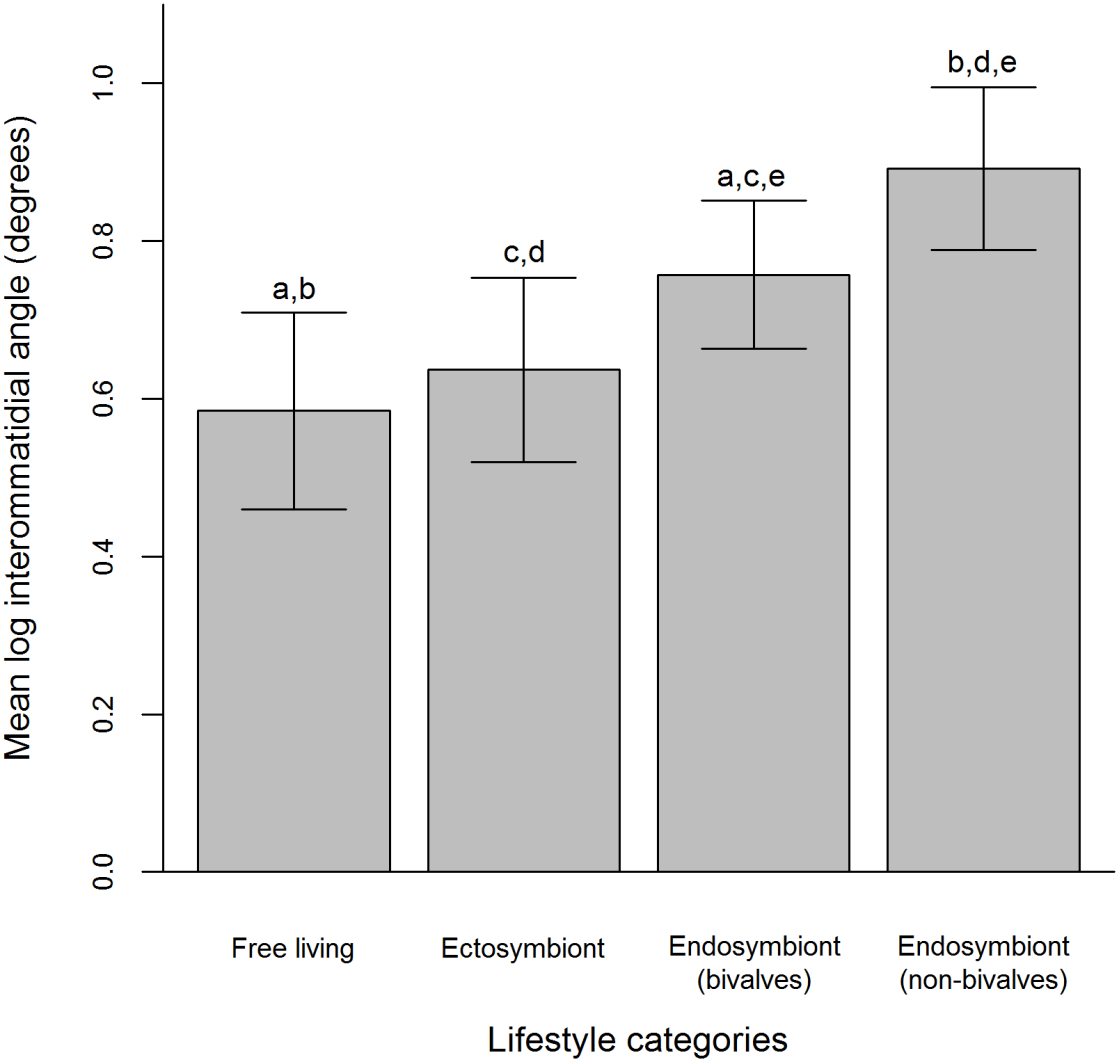
Mean log interommatidial angle for 96 species of Pontoniinae associated with four lifestyle categories. Significant differences (Tukey HSD *P*<0.05) between lifestyle categories were denoted as a, b, c, d and e, with lifestyle categories bearing the same letter being statistically different.

**Figure 7:**
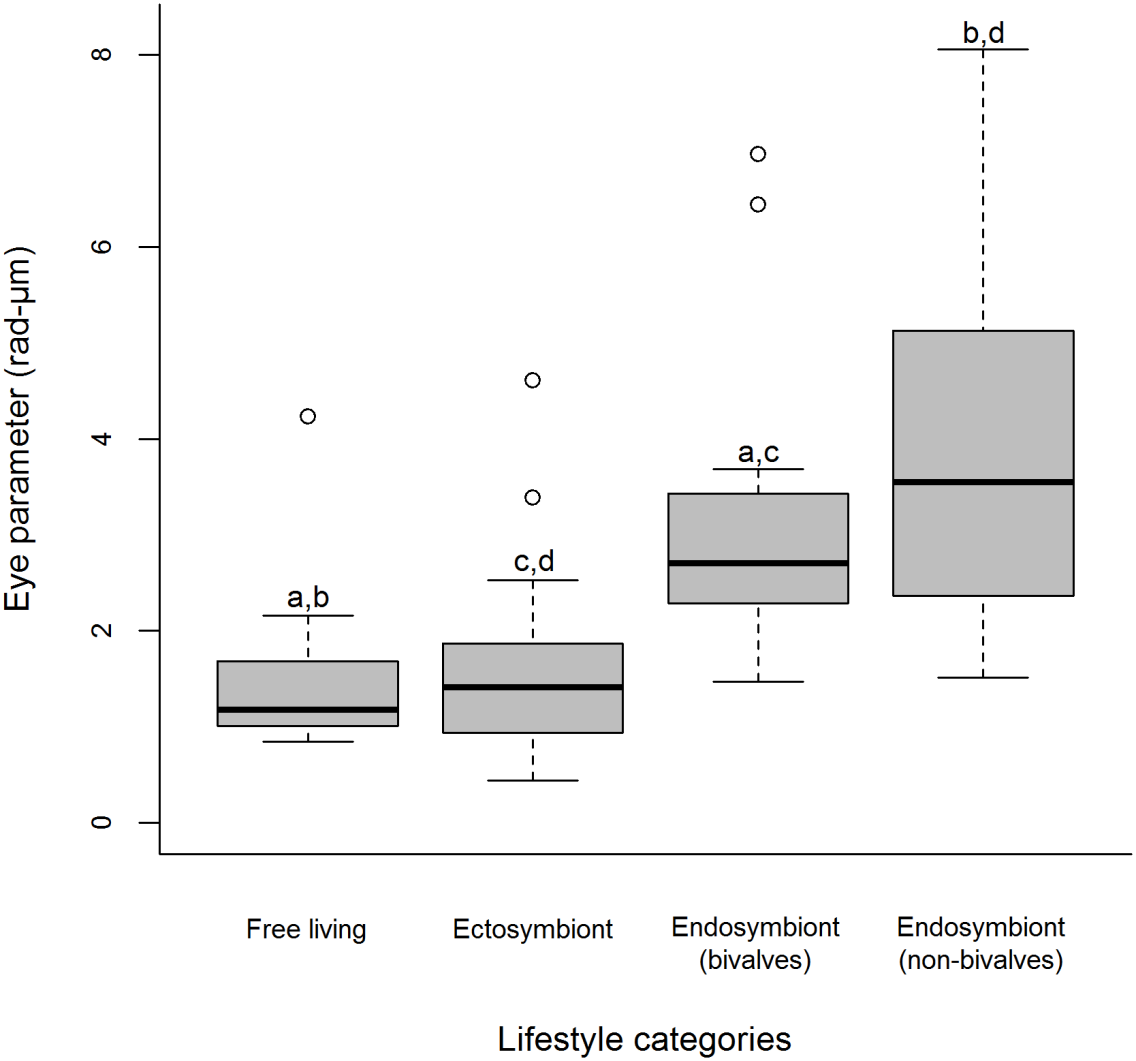
Median eye parameter for 96 species of Pontoniinae associated with four lifestyle categories. Significant differences (Tukey HSD *P*<0.05) between lifestyle categories were denoted as a, b, c, and d, with lifestyle categories bearing the same letter being statistically different.

Interommatidial angles (Δφ) in the shrimps investigated ranged between 2.5° to 11.3°, with significant differences in the mean log^10^ Δφ among the four lifestyle categories (ANOVA, F_3,92_ = 28.40, *P*<0.001). Endosymbionts (non-bivalves) (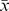 = 0.892, SD±0.10) and endosymbiont (bivalves) (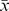 = 0.757, SD±0.09) possess larger Δφ than ectosymbionts (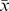 = 0.637, SD±0.12) and free-living individuals (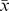 = 0.585, SD±0.12). However, Δφ are smaller in endosymbiont (bivalves) than their non-bivalve counterparts. No differences were found between free-living and ectosymbiotic shrimps (Tukey *P* = 0.05) (Fig. 6). The EPs calculated for all species by the 4 lifestyle categories ranged from between 0.44 to 8.05 rad-µm. Significant differences were found among the EPs of the lifestyle categories (Kruskal Wallis, H (adjusted for ties) = 41.2, df = 3, *P*<0.001, *Post hoc* pairwise comparisons *P* = 0.05) with free living and ectosymbiotic species having significantly smaller EPs than both endosymbiotic lifestyle categories. On average, EPs appear to increase as the lifestyle of the shrimps become more endosymbiotic (Fig. 7).

**Figure 8:**
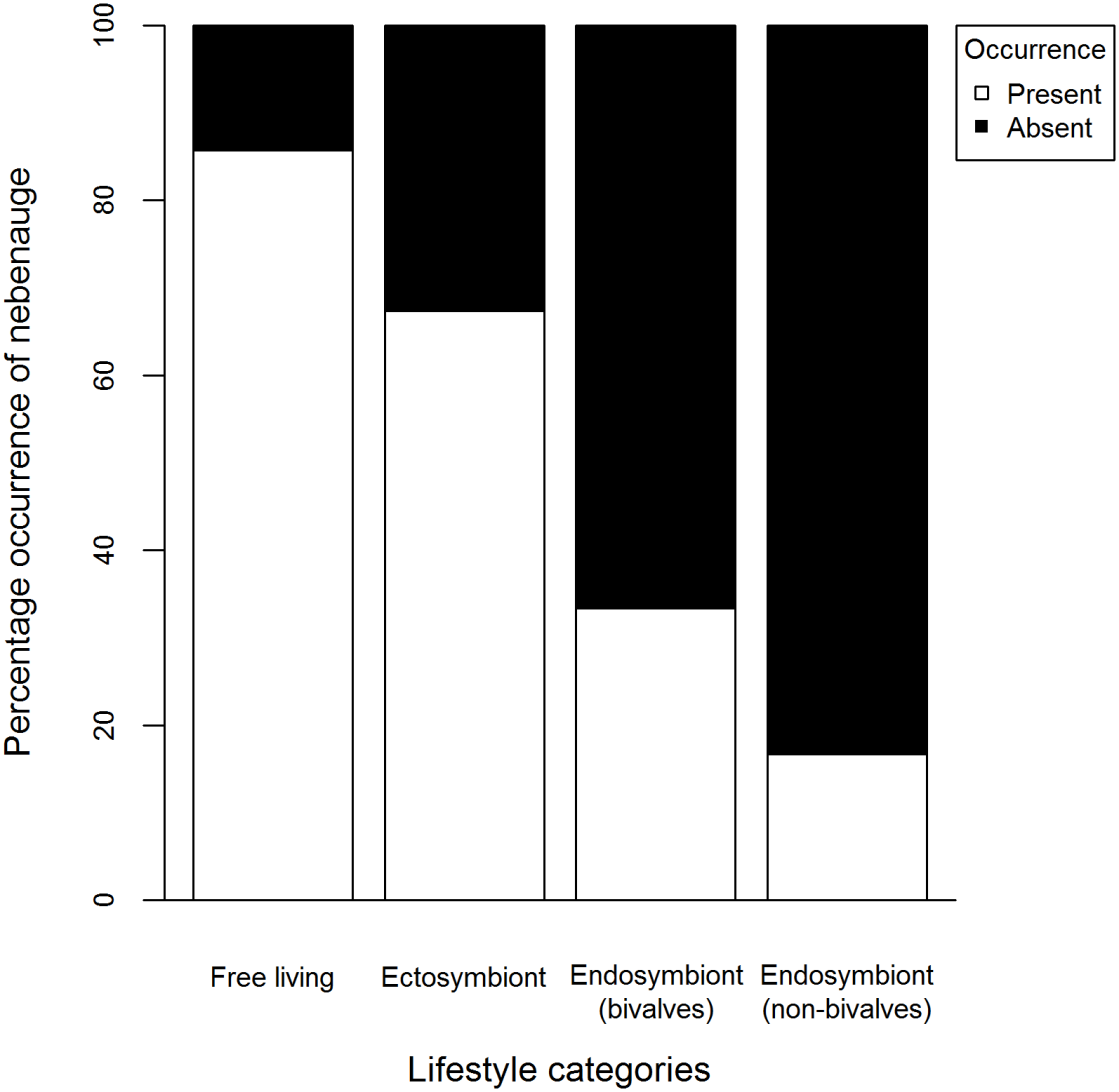
Percentage occurrence of the nebenauge for 96 species of Pontoniinae associated with four lifestyle categories.

Significant differences were also observed in the presence/absence of the nebenauge among shrimps of different lifestyle categories (Chi-squared test, χ^2^ = 21.54, df = 3, *P*<0.001). Nebenauge presence is infrequently associated with the endosymbiotic (non-bivalve) lifestyle, although slightly more frequent in endosymbiotic (bivalve) shrimps. In contrast, high frequencies of free-living species have them, with an equal presence/absence in ectosymbiotic species (Fig. 8). However, when the nebenauge is present there is no difference in relative size between Pontoniinae species of the different lifestyle categories (ANOVA, F_3,50_ = 1.17, *P* = 0.330).

## Discussion

The examination of gross eye morphology of pontoniid shrimps revealed extensive morphological variations, but equally surprising similarities of unrelated species among and between the herein utilised, broad, lifestyle categories. The eyes of free-living and ectosymbiotic species, with distinctly different habitats, were found to be very similar, with the exception of nebenauge occurrence. However, the eyes of endosymbionts are fundamentally different from both free-living and ectosymbionts in terms of relative eye diameter (ED), total eye and stalk length (TEASL), interommatidial angle (Δφ) and nebenauge occurrence. Additionally, there are also significant differences between species classed as bivalve and non-bivalve endosymbiont associates, with the exception of relative TEASL. These similarities and differences underlie their overall visual capabilities in terms of resolution and sensitivity.

Pontoniine shrimps, as with other organisms [75], [76], exhibit a log-log relationship between body size and eye diameter. However, our results demonstrate differences in relative eye diameter (adjusted to body size) among the four lifestyle categories. Free-living and ectosymbionts possess significantly larger eyes relative to body size than endosymbiont (bivalves) while only free-living species possess significantly larger eyes than endosymbionts (non-bivalves). Species with larger compound eyes and smaller facets, as recorded for free-living and ectosymbiotic species, potentially possess more enhanced visual acuity than those with small eyes and larger facets due to the possible increase in photoreceptors [65], [77], smaller interommatidial angles and eye parameters. The combination of these factors observed in Pontoniinae imply that both free-living and ectosymbiotic species possess better visual acuity than their endosymbiotic counterparts. Previous research has also suggested that the eye size of benthic crustaceans can reflect their feeding behaviour, with small eyed species with lower resolution possibly favouring filter feeding, grazing or consuming detritus [49] or perhaps in the case of Pontoniinae, evidence of a parasitic lifestyle.

Species such as *Cuapetes grandis*, *Palaemonella holmesi* and *C. elegans*, categorised as free-living, possess some of the largest relative EDs of the shrimps examined. Larger EDs potentially increase the number of ommatidial units contained within the eyes. The more ommatidia the better the overall resolution (visual acuity) of the eye. Larger EDs for these species coupled with their relatively small facet diameters (FD), potentially increase the number of ommatidia in addition to reducing Δφ, resulting in higher visual acuity increasing the complexity of the visual surroundings they can interpret. Species that commonly have small Δφ and large eyes are often predators, requiring good resolution to detect their prey. These free-living species are generally considered to be scavengers, although some such as *Palaemonella rotumana* and *P. spinulata* are possibly micro-predators [78]. These would thus require reasonable resolution for both prey detection and predator avoidance, as herein demonstrated by eye morphology. Ectosymbionts, such as *Ancylomenes pedersoni* and *Periclimenes yucatanicus* are fish cleaning shrimp that are commonly associated with anemones [79]. Species such as these signal to “clients” by swaying their bodies and waving their antennal flagella with individuals aggressively competing for the best location on the anemone [6]. These behaviours may require better resolution to identify clients in addition to selecting the most favourable location on a host, similar to the visual requirements of free-living species. These shrimps may also be less obligatory and more plastic in host acceptance to locate the best anemones to attract clients, whilst species that are not cleaners are considered more host specific [80]. In general, ectosymbiotic shrimps inhabit more exposed environments than endosymbionts increasing predation risk resulting in a greater requirement for larger eyes and a reasonable level of resolution. Endosymbiotic shrimps however potentially benefit little from good visual acuity as many, but not all, of these shrimp species spend their lives mainly within the confines of their host. The smaller relative eye diameter in all endosymbionts is similar to that of the eye reduction observed in crustacean stygobionts [54], [81]. However, the dramatic reduction observed in stygobionts has rendered the eyes non-visually functional, which is not the case for the endosymbiotic species herein examined. Small compound eyes have two main constraints; fewer ommatidia and relatively larger FDs the combination of which leads to increased interommatidial angles (Δφ) and poorer resolution [65]. Endosymbiont (non-bivalves) species such as *Onycocaris quadratophthalma*, *Periclimenaeus maxillulidens* and *Pontonia panamica* have relatively large FDs (often unorganised in appearance, pers. obs.) and small EDs, even in absolute terms, resulting in larger Δφ and the largest within Pontoniinae, and thus poorer resolution. However larger FDs do have a benefit to shrimps occupying these habitats, as in light adapted eyes increased FD results in an increased photon catch per ommatidia and thus improved sensitivity. Sponge-dwelling endosymbionts of the genera *Onycocaris*, *Periclimenaeus* and *Typton* have traditionally been considered as obligatory symbionts of sponges and ascidians, but -some at least- are now considered parasites, feeding on spongin and spicules [13]. This move to parasitism would negate the requirement for high visual acuity and investment in neural tissue, assuming that the individual shrimp spend their entire life cycle inside their host. A similar pattern can be seen in endosymbiont (bivalves) species however although relative eye sizes were smallest in this category this result is possibly not a true reflection of the impact of symbiosis on eye morphology, due to the unusually large body size observed for these symbionts. Additionally Δφ for endosymbionts (non-bivalves) are significantly larger than free-living or ectosymbionts with endosymbiont (bivalves) forming an almost intermediary group.

Small variations in external morphology can have major implications for the visual ecology of these shrimps as compound eyes are unable to maximize sensitivity and visual acuity simultaneously [64]. It is interesting that endosymbionts (bivalves) form an intermediary group for Δφ between ectosymbionts and endosymbiont (non-bivalves), as this would suggest that their visual acuity is better than endosymbiont (non-bivalves) but not as high as ectosymbionts. A possible explanation is linked to their sexual behaviour, as demonstrated in some species. Co-habiting males of *Pontonia mexicana* in *Pinna carnea* are known to move host in search of reproductive females [3], such mate-searching would require a higher level of acuity to avoid predation and possibly host location if visual cues are used, this should be possible given the depth range of these species are mostly within 100 m (Appendix S1). However, the sensory basis of this search behaviour has not been studied and the potential role of pheromones released from the receptive females is not known. A congeneric species, *Pontonia margarita*, that lives in heterosexual pairs in *Pinctada mazatlanica*, is also known to roam between hosts, but less infrequently [82]. In contrast a species from a phylogenetically unrelated genus also dwelling in bivalves, *Paranchistus pycnodontae*, appears to live in stable, long term heterosexual pairs in *Pteria penguin*, with males displaying no roaming activity [38].

Non-bivalve endosymbionts possess an extremely reduced eyestalk and eyes, with in some instances flattened corneas and a disorganised square facet array, which is especially evident in sponge dwelling species, such as *Onycocaris quadratophthalma*. The fact that endosymbionts still possess eyes may reflect 1) how long these species have been endosymbionts or 2) that their eyes continue to be beneficial. If that is the case then why do these shrimps need them and what could they be using them for? Pontoniinae species that spend the majority of their lives within their host experience reduced light conditions from that of the external surroundings. The eyes of these species are adapted to maximize the light availability by having larger facets and larger interommatidial angles. Some ascidian symbionts, such as *Ascidonia flavomaculata*, are also known to leave the confines of their host in search of food and return to the ascidian for refuge [83]. However, Baeza & Díaz-Valdés [1] also found that individuals, especially males, will leave the ascidian in search of a mate, although females possibly move around during the summer in search of larger hosts. This mate searching behaviour may also occur in *Ascidonia miserabilis*, however this is yet to be confirmed [84]. These behaviours suggest that these species are less obligate and more facultative in their symbiotic relationship which is reflected in their eye morphology by smaller interommatidial angles and eye parameters therefore potentially increased visual acuity. In dark environments the eye parameter (EPs) has been shown to increase [73]. The maximum EP recorded for living species is of 44 rad-µm however, larger values of 100 rad-µm have been found in fossil trilobites [85]. Nocturnal or deep sea species often have EPs ranging to 20 rad-µm whilst species from well-lit environments can possess an EP as small as 0.3 rad-µm [86]. The significantly smaller EP results observed in free-living and ectosymbiotic species (1.2 rad-µm and 1.4 rad-µm respectively) suggest that these shrimps are adapted to brighter habitats than their endosymbiotic counterparts, both bivalve (2.7 rad-µm) and non-bivalves (3.6 rad-µm). As these species do not travel at high speeds, which would require larger Δφ to sustain the resolving power of the eye due to the angular velocity, the high EPs of the endosymbionts can probably be attributed to these shrimps adapting to darker conditions rather than the sampling frequency of the eye. As a consequence species with higher EPs sacrifice resolution in favour of higher sensitivity [72]. It is likely that these differences are driven by a balance between the relatively high physiological cost of eyes [87] and their likely benefits. Although such differences can be seen in our data, the reason why these species, especially sponge-dwellers, have eyes and what they are using them for remains unclear.

Ugolini and Borgiolo [67] stated difficulties in associating nebenauge position with ecology or behaviour of shrimps. However, our results reflect a pattern in the presence or absence of nebenauge among the four ecological lifestyles categories, suggesting that basic ecology may influence the occurrence in Pontoniinae. Itaya [88] suggested that the nebenauge is a type of small compound eye responding to light levels, but with a different role to the rest of the eye. Histological sections from mesopelagic shrimps [46] revealed functioning ommatidia that were structurally different from the adjoining cornea. Whilst the presence of optics suggests that differences in light intensity can be detected it seems unlikely that this region would be able to resolve an image. The results for Pontoniinae show a decrease in nebenauge presence in relation to increasing divergence from a free-living mode of life. The notion that nebenauge may be used for light orientation and/or light detection is supported by these results where nebenauge in free-living species could be advantageous but would be superfluous for endosymbionts (non-bivalves) where phototactic behaviour would be minimal. Bruce [89] described how bivalve symbionts can be separated into three groups based on morphology. It is currently unclear if these groups represent phylogenetic clades or the result of convergent evolution and independent host invasions. The groups are herein used, but only as an indication of gross bauplan morphology, and not an indication of phylogeny. The first group contains the genera *Anchistus*, *Neoanchistus* and *Paranchistus* with this group considered the most primitive and less specialised than other bivalve associates [89]. The presence of a well-developed dentate rostrum as well as antennal and hepatic spines supports this notion. Interestingly, these same genera are some of the few bivalve symbionts where the nebenauge is present in all of these genera. The second group includes *Platypontonia* and *Pontonia*; these genera are considered more specialised to their hosts, primarily linked to their unarmed short rostra and the finer details of their ambulatory pereiopods. Within this group the nebenauge is occasionally present in some species such as *Pontonia margarita*. However the third group, *Conchodytes* and *Chernocaris* (the latter now considered a synonym of *Conchodytes*, see [90]), are most specialised and within this group the nebenauge is absent from all genera. This direction in the absence or presence of the nebenauge in bivalve-associated Pontoniinae does appear to indicate an increased level of specialisation for these species, supporting Bruce’s [89] scheme of evolutionary relationships for these genera.

Ellers [91] suggested that ecological interactions can impact the expression of traits, as demonstrated here in the occurrence of nebenauge, eye and stalk size in addition to variations in interommatidial angles and eye parameters among lifestyle categories. It is likely that in some endosymbiotic species stalked, well developed hemispherical eyes with high acuity are no longer of benefit. Although the categorisation of associations of shrimps with hosts into four broad classes, endosymbiont (bivalves), endosymbiont (non-bivalves), ectosymbionts and free-living, is undoubtedly a gross simplification and is not based on the emerging complex spectrum of symbiotic behaviours, such as host selection, usage or switching in some genera (e.g. [3]), external characteristics in compound eye design of Pontoniinae are clearly linked to their lifestyle and hence their fundamental ecological position. Simplification and modification of eyes in these shrimps thus demonstrates how they have adapted and evolved over time to environmental pressures and conditions, yet it is surprising to note the degree of morphological plasticity within the same subfamily. Not only has eye morphology been seen to be a phenotypic (physical and physiological variation within a population) trait among terrestrial species such as flies [92], [93], mosquitoes [69] and beetles [94], evidence here suggests that these features display evolutionary plasticity under relatively specific conditions. The fact that Pontoniinae are symbiotic with a diverse array of taxa may have acted as a driver for the variability in optical structures observed, as it clearly has done for other aspects of their bauplan. These results highlight the tensions between ecology, physiology and systematics in the evolution of compound eyes.

## Supporting Information

Figure S1 **Photograph of** ***Anchistus custos*** **illustrating measurement taken for post-orbital carapace length.**
(TIF)

Figure S2 **Photograph of** ***Palaemonella holmesi*** **illustrating external measurements taken from the eye including eyestalk length (ESL), total eye and stalk length (TEASL), eye diameter (ED), nebenauge diameter (ND) and facet diameter (FD).**
(TIF)

Appendix S1 **Species list of Pontoniinae used within this investigation including Oxford University Museum of Natural History (OUMNH) catalogue numbers, assigned lifestyle category and host association [14]** **.**
(DOC)
