# Supplementary figures and images for: Linking Eye Design with Host Symbiont Relationships in Pontoniine Shrimps (Crustacea, Decapoda, Palaemonidae)
Source: PLoS One. 2014 Jun 20;9(6):e99505. doi: 10.1371/journal.pone.0099505 (PMC4064969; doi:10.1371/journal.pone.0099505)

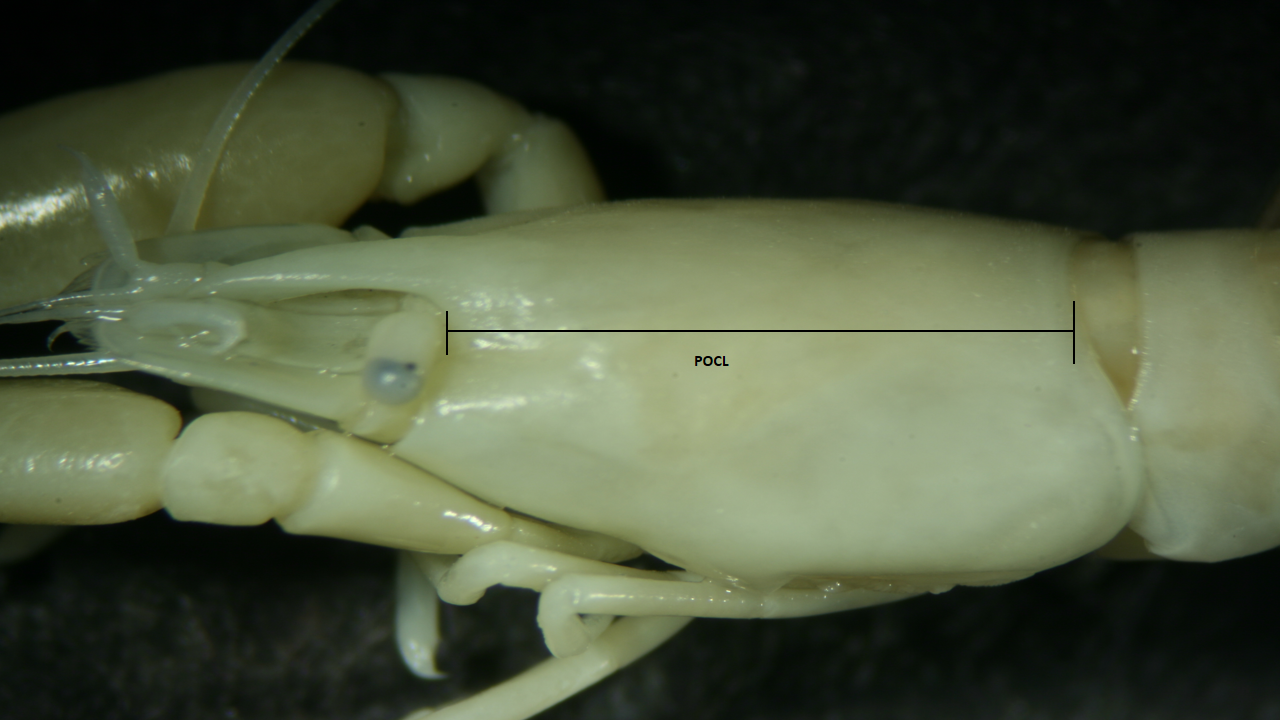

Supplement: Figure S1 — Photograph of Anchistus custos illustrating measurement taken for post-orbital carapace length. (TIF) [file pone.0099505.s001.tif]

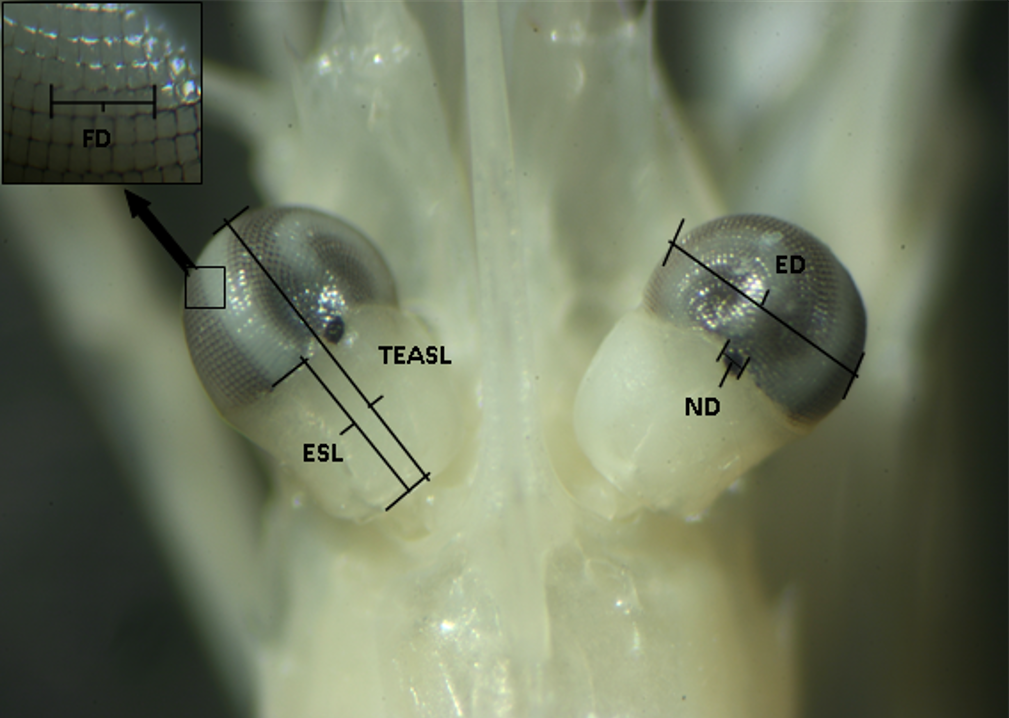

Supplement: Figure S2 — Photograph of Palaemonella holmesi illustrating external measurements taken from the eye including eyestalk length (ESL), total eye and stalk length (TEASL), eye diameter (ED), nebenauge diameter (ND) and facet diameter (FD). (TIF) [file pone.0099505.s002.tif]
