# Supplementary material for: Linking Eye Design with Host Symbiont Relationships in Pontoniine Shrimps (Crustacea, Decapoda, Palaemonidae)
Source: PLoS One. 2014 Jun 20;9(6):e99505. doi: 10.1371/journal.pone.0099505 (PMC4064969; doi:10.1371/journal.pone.0099505)
Supplement: Appendix S1 — Species list of Pontoniinae used within this investigation including Oxford University Museum of Natural History (OUMNH) catalogue numbers, assigned lifestyle category and host association [14] . (DOC) [file pone.0099505.s003.doc]

| **Species** | **Catalogue number** | **Lifestyle category** | **Host** | **Depth (m)** |
| --- | --- | --- | --- | --- |
| *Anchistus custos* | OUMNH.ZC.2002-16-0003 | Endosymbiont (bivalves) | Bivalvia | 0 -110 |
| *Ancylomenes holthuisi* | OUMNH.ZC.2010-12-0070 | Ectosymbiont | Actiniaria | 0-70 |
| *Ancylomenes pedersoni* | OUMNH.ZC.2004-22-015 | Ectosymbiont | Actiniaria | 0-40 |
| *Ancylomenes tosaensis* | OUMNH.ZC.2003-30-0013 | Ectosymbiont | Actiniaria | 30-150 |
| *Ancylomenes venustus* | OUMNH.ZC.2010-12-0066 | Ectosymbiont | Actiniaria | 0-30 |
| *Balssia gasti* | OUMNH.ZC.2002-09-001 | Ectosymbiont | Scleractinia | 5-120 |
| *Brucecaris tenuis* | OUMNH.ZC.2005-09-0024 | Ectosymbiont | Crinoidea | 0-30 |
| *Cainonia medipacifica* | OUMNH.ZC.2010-02-0053 | Endosymbiont (bivalves) | Bivalvia | 0-10 |
| *Conchodytes biunguiculatus* | OUMNH.ZC.2009-10-0001 | Endosymbiont (bivalves) | Bivalvia | 0-50 |
| *Conchodytes meleagrinea* | OUMNH.ZC.2005-09-0025 | Endosymbiont (bivalves) | Bivalvia | 0-30 |
| *Conchodytes nipponensis* | OUMNH.ZC.2010-04-0002 | Endosymbiont (bivalves) | Bivalvia | 0-20 |
| *Conchodytes placunae* | OUMNH.ZC.2010-03-0011 | Endosymbiont (bivalves) | Bivalvia | 5-20 |
| *Coralliocaris superba* | OUMNH.ZC.2005-09-0027 | Ectosymbiont | Scleractinia | 0-60 |
| *Coralliocaris viridis* | OUMNH.ZC.2010-14-0024 | Ectosymbiont | Scleractinia | 0-20 |
| *Cuapetes americanus* | OUMNH.ZC.2005-10-0040 | Free living | Free living | 0-65 |
| *Cuapetes andamanensis* | OUMNH.ZC.2009-09-0025 | Free living | Free living | 0-80 |
| *Cuapetes elegans* | OUMNH.ZC.2009-18-0034 | Free living | Free living | 0-60 |
| *Cuapetes ensifrons* | OUMNH.ZC.2007-22-0017 | Free living | Free living | 0-40 |
| *Cuapetes grandis* | OUMNH.ZC.2006-20-006 | Free living | Free living | 0-30 |
| *Cuapetes kororensis* | OUMNH.ZC.2010-12-0019 | Ectosymbiont | Scleractinia | 5-20 |
| *Cuapetes seychellensis* | OUMNH.ZC.2010-12-0071 | Free living | Free living | 0-75 |
| *Cuapetes tenuipes* | OUMNH.ZC.2010-12-0021 | Free living | Free living | 0-160 |
| *Dactylonia okai* | OUMNH.ZC.2010-14-0033 | Endosymbiont (non-bivalves) | Ascidiacea | 10-90 |
| *Fennera chacei* | OUMNH.ZC.2010-14-0035 | Ectosymbiont | Scleractinia | 0-40 |
| *Hamodactylus boschmai* | OUMNH.ZC.2010-12-0016 | Ectosymbiont | Gorgonacea | 0-45 |
| *Hamopontonia corallicola* | OUMNH.ZC.2010-12-0036 | Ectosymbiont | Scleractinia | 0-20 |
| *Harpiliopsis beaupressi* | OUMNH.ZC.2005-09-0010 | Ectosymbiont | Scleractinia | 0-45 |
| *Harpiliopsis spinigera* | OUMNH.ZC.2010-14-0002 | Ectosymbiont | Scleractinia | 0-20 |
| *Harpilius bayeri* | OUMNH.ZC.2005-09-0013 | Ectosymbiont | Scleractinia | 0-10 |
| *Harpilius consobrinus* | OUMNH.ZC.2005-09-0011 | Ectosymbiont | Scleractinia | 0-25 |
| *Holthuisaeus bermudensis* | OUMNH.ZC.2005-10-0032 | Endosymbiont (non-bivalves) | Porifera | 0-25 |
| *Ischnopontonia lophos* | OUMNH.ZC.2005-09-0034 | Ectosymbiont | Scleractinia | 0-20 |
| *Laomenes amboinensis* | OUMNH.ZC.2010-12-0064 | Ectosymbiont | Crinoidea | 0-40 |
| *Laomenes ceratophthalmus* | OUMNH.ZC.2002-24-0064 | Ectosymbiont | Crinoidea | 0-35 |
| *Laomenes cornutus* | OUMNH.ZC.2002-24-0077 | Ectosymbiont | Crinoidea | 10-20 |
| *Laomenes nudirostris* | OUMNH.ZC.2004-13-0001 | Ectosymbiont | Crinoidea | 5-35 |
| *Manipontonia psamathe* | OUMNH.ZC.2010-02-0016 | Ectosymbiont | Gorgonacea | 0-110 |
| *Neoanchistus nasalis* | OUMNH.ZC.2002-16-004 | Endosymbiont (bivalves) | Bivalvia | 0-15 |
| *Neopontonides chacei* | OUMNH.ZC.2005-10-0099 | Ectosymbiont | Gorgonacea | 0-15 |
| *Neopontonides beaufortensis* | OUMNH.ZC.2004-17-0003 | Ectosymbiont | Gorgonacea | 0-20 |
| *Odontonia katoi* | OUMNH.ZC.2010-12-0022 | Endosymbiont (non-bivalves) | Ascidiacea | 0-50 |
| *Onycocaris quadratophthalma* | OUMNH.ZC.2010-14-0006 | Endosymbiont (non-bivalves) | Porifera | 0-10 |
| *Onycocaris* sp. | OUMNH.ZC.2010-14-0007 | Endosymbiont (non-bivalves) | Porifera | 0-20 |
| *Orthopontonia ornata* | OUMNH.ZC.2010-12-0001 | Endosymbiont (non-bivalves) | Porifera | 5-20 |
| *Palaemonella holmesi* | OUMNH.ZC.2007-13-0018 | Free living | Free living | 0-130 |
| *Palaemonella pottsi* | OUMNH.ZC.2010-12-0002 | Ectosymbiont | Crinoidea | 0-40 |
| *Palaemonella spinulata* | OUMNH.ZC.2010-12-0035 | Free living | Free living | 0-80 |
| *Paranchistus pycnodontae* | OUMNH.ZC.2010-02-0002 | Endosymbiont (bivalves) | Bivalvia | 0-10 |
| *Periclimenaeus ascidiarum* | OUMNH.ZC.2007-14-0006 | Endosymbiont (non-bivalves) | Ascidiacea | 0-75 |
| *Periclimenaeus bredini* | OUMNH.ZC.2009-22-0002 | Endosymbiont (non-bivalves) | Porifera | 0-5 |
| *Periclimenaeus caraibicus* | OUMNH.ZC. 2005-10-0033 | Endosymbiont (non-bivalves) | Porifera | 0-20 |
| *Periclimenaeus hecate* | OUMNH.ZC.2002-16-0002 | Endosymbiont (non-bivalves) | Ascidiacea | 0-65 |
| *Periclimenaeus maxillulidens* | OUMNH.ZC.2007-14-0007 | Endosymbiont (non-bivalves) | Porifera | 0-55 |
| *Periclimenaeus orbitocarinatus* | OUMNH.ZC.2010-14-0021 | Endosymbiont (non-bivalves) | Ascidiacea | 10-25 |
| *Periclimenaeus storchi* | OUMNH.ZC.2010-12-0010 | Endosymbiont (non-bivalves) | Ascidiacea | 5-15 |
| *Periclimenella spinifera* | OUMNH.ZC.2010-12-0017 | Free living | Free living | 0-40 |
| *Periclimenes colemani* | OUMNH.ZC.2010-03-0001 | Ectosymbiont | Echinoidea | 10-25 |
| *Periclimenes commensalis* | OUMNH.ZC.2010-12-0067 | Ectosymbiont | Crinoidea | 0-50 |
| *Periclimenes gonioporae* | OUMNH.ZC.2010-14-0011 | Ectosymbiont | Scleractinia | 0-10 |
| *Periclimenes harringtoni* | OUMNH.ZC.2013-05-0063 | Ectosymbiont | Porifera | 10-20 |
| *Periclimenes incertus* | OUMNH.ZC.2010-12-0024 | Ectosymbiont | Porifera | 0-55 |
| *Periclimenes inornatus* | OUMNH.ZC.2010-12-0026 | Ectosymbiont | Actiniaria | 0-35 |
| *Periclimenes* cf *iridescens* |  | Ectosymbiont | Gorgonacea | 0-180 |
| *Periclimenes kempi* | OUMNH.ZC.2010-12-0027 | Ectosymbiont | Gorgonacea | 0-25 |
| *Periclimenes madreporae* | OUMNH.ZC.2010-14-0012 | Ectosymbiont | Scleractinia | 0-15 |
| *Periclimenes ornatus* | OUMNH.ZC.2005-09-0037 | Ectosymbiont | Actiniaria | 0-20 |
| *Periclimenes patae* |  | Ectosymbiont | Gorgonacea | 0-25 |
| *Periclimenes pauper* | OUMNH.ZC.2005-10-0051 | Ectosymbiont | Gorgonacea | 0-15 |
| *Periclimenes perryae* | OUMNH.ZC.2005-10-0056 | Ectosymbiont | Ophiuroidea | 5-20 |
| *Periclimenes rathbunae* | OUMNH.ZC.2005-10-0055 | Ectosymbiont | Actiniaria | 0-20 |
| *Periclimenes scriptus* | OUMNH.ZC.2007-24-0013 | Ectosymbiont | Actiniaria | 0-120 |
| *Periclimenes siankaanensis* | OUMNH.ZC.2008-22-0001 | Free living | Free living | 0-10 |
| *Periclimenes soror* | OUMNH.ZC.2010-12-0012 | Ectosymbiont | Asteroidea | 0-40 |
| *Periclimenes* cf *antipathophilus* |  | Ectosymbiont | Gorgonacea | 15-50 |
| *Periclimenes yucatanicus* | OUMNH.ZC.2008-14-0029 | Ectosymbiont | Actiniaria | 0-30 |
| *Phycomenes indicus* | OUMNH.ZC.2010-12-0069 | Free living | Free living | 0-60 |
| *Phycomenes zostericola* | OUMNH.ZC.2009-09-0023 | Free living | Free living | 0-5 |
| *Platypontonia hyotis* | OUMNH.ZC.2010-02-0001 | Endosymbiont (bivalves) | Bivalvia | 0-30 |
| *Pontonia margarita* | OUMNH.ZC.2006-10-0012 | Endosymbiont (bivalves) | Bivalvia | 0-65 |
| *Pontonia mexicana* | OUMNH.ZC.2007-20-0121 | Endosymbiont (bivalves) | Bivalvia | 0-25 |
| *Pontonia panamica* | OUMNH.ZC.2007-13-0030 | Endosymbiont (non-bivalves) | Ascidiacea | 0-1 |
| *Pontonia pinnophylax* | OUMNH.ZC.2008-11-0081 | Endosymbiont (bivalves) | Bivalvia | 0-150 |
| *Pontonides loloata* | OUMNH.ZC.2010-12-0012 | Ectosymbiont | Gorgonacea | 5-115 |
| *Pontoniopsis comanthi* | OUMNH.ZC.2010-12-0018 | Ectosymbiont | Crinoidea | 0-35 |
| *Pseudopontonides principis* |  | Ectosymbiont | Gorgonacea | 10-70 |
| *Rapipontonia galene* | OUMNH.ZC.2010-12-0073 | Ectosymbiont | Hydrozoa | 0-30 |
| *Rapipontonia platalea* | OUMNH.ZC.2009-27-0002 | Ectosymbiont | Hydrozoa | 10-40 |
| *Thaumastocaris streptopus* | OUMNH.ZC.2008-08-0002 | Ectosymbiont | Porifera | 5-145 |
| *Tuleariocaris neglecta* | OUMNH.ZC.2007-04-0001 | Ectosymbiont | Echinoidea | 0-20 |
| *Tuleariocaris zanzibarica* | OUMNH.ZC.2010-03-0003 | Ectosymbiont | Echinoidea | 0-10 |
| *Typton gnathophylloides* | OUMNH.ZC.2004-22-0033 | Endosymbiont (non-bivalves) | Porifera | 20-85 |
| *Typton hephaestus* | OUMNH.ZC.2007-13-0017 | Endosymbiont (non-bivalves) | Porifera | 10-20 |
| *Typton holthuisi* | OUMNH.ZC.2008-11-0068 | Endosymbiont (non-bivalves) | Porifera | 0-5 |
| *Typton tortugae* | OUMNH.ZC.2007-14-0004 | Endosymbiont (non-bivalves) | Porifera | 0-20 |
| *Urocaris longicaudata* | OUMNH.ZC.2008-14-0037 | Free living | Free living | 0-30 |
| *Vir philippinensis* | OUMNH.ZC.2010-12-0003 | Ectosymbiont | Scleractinia | 5-35 |
